# Huge Enhancement of Luminescence from a Coaxial-Like Heterostructure of Poly(3-methylthiophene) and Au

**DOI:** 10.3390/polym10040414

**Published:** 2018-04-09

**Authors:** Bo-Hyun Kim, Hojin Lee, Do Hyoung Kim, Seokho Kim, Jinho Choi, Gil Sun Lee, Dong Hyuk Park, Sunjong Lee

**Affiliations:** 1 Korea Institute of Industrial Technology, Chenan, Chungnam 31056, Korea; bohkim@kitech.re.kr; 2 Department of Applied Organic Materials Engineering, Inha University, Incheon 22212, Korea; hojin@inha.edu (H.L.); gg1236@inha.edu (D.H.K.); seokho@inha.edu (S.K.); jinho@inha.edu (J.C.); 3 Department of General Education, Kookmin University, Seoul 02707, Korea; 4 Department of Chemical Engineering, Inha University, Incheon 22212, Korea

**Keywords:** P3MT, photoluminescence, heterostructure, surface plasmon resonance, corss-junction

## Abstract

Recently, the light-matter interaction at nanoscale has attracted great interest from physicists, chemists and material scientists, as it gives peculiar optical properties that couldn’t be observed at the bulk scale. The synthesis and characterization of organic-inorganic heterostructures forming quantum dots, nanowires or nanotubes provide opportunities to understand their photophysical mechanism and to apply optoelecronic devices. Herein, we report a huge enhanced luminescence in a coaxial-like heterostructured poly (3-methylthiophene) (P3MT) with Au. We electrochemically synthesized P3MT nanowires (NWs) on a nanoporous template, and sequentially deposited Au on the surface of P3MT NWs. The diameter of heterostructured P3MT/Au NWs was about 200 nm, where the cladding-shape Au were about 10 nm. The visible range absorbance, with two new absorption peaks of P3MT/Au NWs, was significantly increased compared with that of P3MT NWs. Accordingly, the photoluminescence (PL) of a P3MT/Au NW was enormously increased; up to 170 times compared to that of P3MT NWs. More interestingly, an unexpected enhancement of PL was observed from cross-junction point of P3MT/Au NWs. The abnormal PL properties of P3MT/Au NWs were attributed to the charge transfer and the surface plasmon resonance between the cladding-shape Au and the core-shape P3MT, which resulted in the enhanced quantum yield. This incites us to reconsider the light-matter interaction in polymer-metal hybrid structures applicable for high-performance optoelectronic devices.

## 1. Introduction

Nano-optics is an emerging field of research that is attracting great interest from physicists, chemists and material scientists, as it provides extraordinary photophysical properties that have not been considered in the bulk scale. Due to the rapid development of nanoscience and nanotechnology, light-matter interactions such as plasmonics, photonic crystals, fluorescence resonance energy transfer, etc. can be studied on a molecular level with sub-wavelength resolution [1,2,3,4,5]. For decades, countless organic, inorganic, and hybridized nano-structures such as quantum dots, nanowires, nanotubes and core-shell particles have been successfully synthesized [6]. These nanomaterials show the extraordinary nano-optical phenomena that depend on shape, size and dimensionality [7,8]. For example, the light-emitting conjugated polymers such as poly thiophenes and their derivative series have attracted intensive notice not only due to their fundamental photophysical mechanism but also for highly efficient light-emitting diodes, photovoltaics, and ultrasensitive chemical and biological sensors [9,10,11,12,13,14,15].

The surface-induced Raman resonance and the surface plasmon resonance are representative research areas that are leading the progress in various optical technologies with applications in opto-electronics, bio-optices and optical sensors [16,17,18]. For example, graphene surface-enhanced resonance Raman scattering showed a Raman signal intensity that was increased by up to ~100 times compared to that on bare substrate [19,20]. They also demonstrated the picomolar sensitive and molecular selective Raman scattering characteristics on graphene substrates. The molecular photoluminescence (PL) enhancement on thin film or/and nanostructured metal has been known for several decades [21,22], although the exact opposite phenomenon was simultaneously shown, whereby the PL was quenched due to the resonant energy transfer within a distance of a few nanometer [23,24]. Au nanostructures have been extensively studied due to their importance in fundamental scientific research and potential industrial application; they exhibit strong surface plasmon resonance in the visible spectrum due to the dependence on size and dimensionality [25,26,27]. Specifically, a local electromagnetic (EM) field enhancement in a form of Au based nano-bow-tie or nano gap has recently been studied in the field of optoelectronic devices and photonics, which might contribute to the tuning of luminescence efficiency and color of the developed nano architectures [28,29,30]. Hybrid nanostructures of organic semiconductors with inorganic metals have been synthesized and applied to high quantum-efficiency bio- and chemical sensors [31,32]. This effect is ascribed to the energy transfer effect contributed by a surface plasmon resonance (SPR) coupling. In addition, the development of high-resolution optical microscope systems has opened up exciting new avenues for probing the optical properties of materials, even at the molecular level.

**Figure 1:**
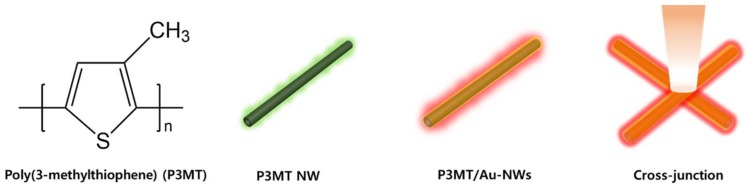
Schematic illustration of molecular structure of poly (3-methylthiophene) (P3MT) and photoluminescence (PL) measurement from P3MT and P3MT/Au nanowires (NWs), and the cross-junction point.

In the previous report, we showed SPR coupling PL enhancement on a single strand of hybrid nanotubes (NTs) consisting of light-emitting polymers and thin metal [33,34]. Herein, we report a huge enhancement of luminescence from coaxial-like nanowires (NWs) made of poly (3-methylthiophene) (P3MT) and Au. Heterostructured NWs with a core diameter of 200 nm and a cladding thickness of 10 nm were fabricated through electrochemical deposition of P3MT sequential Au on the nanoporous template. The photoluminescence characteristics were measured by using a home-made laser confocal microscope (LCM) system from an isolated single strand NW of P3MT and P3MT/Au, and the cross-junction site of P3MT/Au NWs (Figure 1). The PL intensity was increased in the P3MT/Au NWs, where the cross-junction points showed much higher PL intensity than that from the isolated single NW.

## 2. Materials and Methods

**Figure 2:**
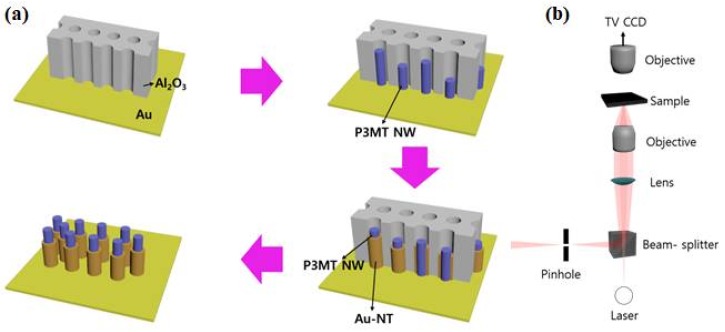
(**a**) Fabrication process of hybrid P3MT/Au NWs using template method; (**b**) PL and Raman measurement using laser confocal microscope (LCM).

Sample preparation: Figure 2a schematically illustrates the synthetic procedure for obtaining the hybrid P3MT/Au NWs. The coaxial-shaped NWs were fabricated by an electrochemical deposition method on the anodic alumina oxide (Al_2_O_3_) nanoporous template with a diameter of ~200 nm and thickness of ~60 μm (Whatman International Ltd., Maidstone, UK). Firstly, Au was thermally deposited onto one side of the Al_2_O_3_ nanoporous template. The Al_2_O_3_ nanoporous template was attached to the working electrode. Then P3MT was electrochemically polymerized and deposited in the channel of nanoporous template. The electrolyte consisted of 3-methylthiophene (3MT) as a monomer (98% purified ones), tetrabutylammonium hexafluorophosphate (TBAPF_6_) as a dopant, and anhydrous acetonitrile (CH_3_CN) as solvent. The molar ratio of monomer to dopant was 5:1. Sequentially, after dissolving the Al_2_O_3_ template only through using the HF solution, P3MT NWs were obtained. The hybrid P3MT/Au NWs were fabricated through sequential galvanostatic deposition of Au on the surface of P3MT NW after polymerization of P3MT. The electrolyte consisted of a solution of Orotemp 24 gold plating solution (TECHNIC INC). Cyclic voltammetry (CV, Bioanalytical Systems Inc., EC Epsilon, West Lafayette, IN, USA) was used for Au coating to the surface P3MT NW. The current density and voltage applied to electrode was ~2.0 mA/cm^2^ and −1.20 ± 0.05 V, respectively, for 10 min. After finishing the NW fabrication, the Al_2_O_3_ template was removed by the HF solution and then the coaxial shaped hybrid P3MT/Au NWs were obtained. More experimental detail are in ref. [35].

Measurement: We visualized the formation and surface morphology of the hybrid P3MT/Au NWs with a scanning electron microscope (SEM, JEOL JSM-5200, LabX, Midland, ON, Canada) and a high-resolution transmission electron microscope (HR-TEM, JEOL JEM-3010, Nanolab Technologies, Milpitas, CA, USA). The luminescence color charge-coupled device (CCD) images of the P3MT NWs and the hybrid P3MT/Au NWs were measured with an AVT Marlin F-033C (*λ_ex_* = 435 nm). In order to compare the luminescence brightness of the various CCD images, we fixed the exposure time with the light at 0.5 s. We measured the nanoscale PL images and spectra for a single unit of the P3MT NWs and of the hybrid P3MT/Au NWs with a laser confocal microscope (LCM) built around an inverted optical microscope (Axiovert 200, Zeiss GmbH, Neubeuern, Germany), as shown in Figure 2b. We used the 488 nm line of Argon ion laser for the LCM PL excitation. The spot size of the focused laser beam on the sample was estimated to be about 190 nm. The laser power incident on the sample and the acquisition time for each LCM PL spectrum were fixed at 35 μW and 1 s, respectively. To prepare the samples for the LCM PL experiments, we homogeneously dispersed the P3MT NWs or the hybrid P3MT/Au NWs in chloroform (CHCl_3_) solution after removal of the Al_2_O_3_ nanoporous template, and they were then ultra-sonicated for 10 s. The dispersed solution of the NWs was drop-cast onto a slide glass and then dried in a vacuum oven (below 10^−3^ Torr) for 30 min at room temperature (RT). The detailed methods for the LCM PL and the LCM Raman experiments have been reported earlier [35].

## 3. Results

### 3.1. Coaxial-Like Hybrid Nanostructure of P3MT/Au

**Figure 3:**
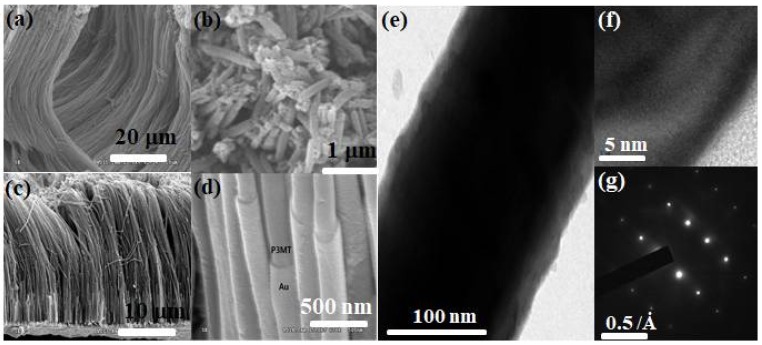
SEM images of the side (**a**,**c**,**d**) and top (**b**) views of P3MT (**a**,**b**) and P3MT/Au NWs (**c**,**d**) after removal of the Al_2_O_3_ nanoporous template; (**e**,**f**) TEM images of P3MT/Au NW; (**g**) SAED pattern from the cladding area of (**e**,**f**).

Figure 3a,b are SEM images showing the side and top view of the P3MT NWs. The NWs have a uniform and a continuous array and wire-like structure with a height of about 60 μm. From the top view, it can be seen that the P3MT NWs are filled such that they have a closed end and a diameter of about 200 nm. Figure 3c,d are a side view and a magnified SEM image of hybrid P3MT/Au NWs. The total length of hybrid P3MT/Au NWs was about 30 μm shorter than P3MT NWs; however, the cladding structure of Au in Figure 3d was clearly observed. From the TEM image (Figure 3e), the total diameter of the hybrid P3MT/Au NW and the cladding thickness of Au were measured to be ~200 nm and ~10 nm, respectively. Figure 3f is the HR-TEM image showing that the lattice fringe with lattice spacing for the Au material was estimated to be ~2.1 A. From a selected area electron diffraction (SAED) pattern of hybrid P3MT/Au NW (Figure 3g), a fine and periodic stripe pattern was clearly observed, suggesting that the cladding is Au with a highly crystalline structure. The SEM and TEM results demonstrate that our P3MT/Au is successfully fabricated as coaxial-like hybrid NWs with a core of P3MT and a cladding of Au.

### 3.2. Photoluminescence (PL) of P3MT and Hybrid P3MT/Au NWs

**Figure 4:**
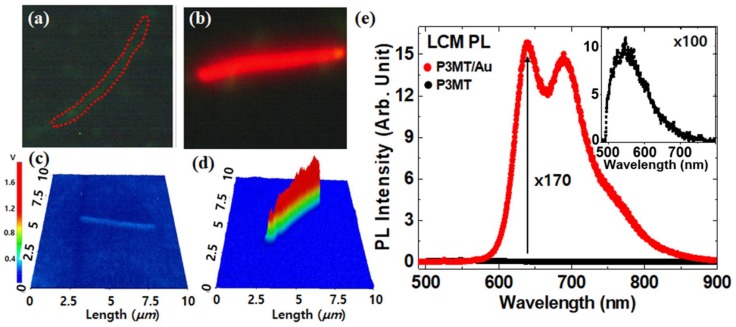
(**a**,**b**) PL CCD images of a P3MT NW (**a**) and a hybrid P3MT/Au NW (**b**); (**c**,**d**) Scanning LCM PL images of an isolated P3MT NW (**c**) and P3MT/Au NW (**d**); (**e**) Comparison of PL spectra between P3MT/Au NW (red line) and P3MT NW (black line) Inset: the magnified PL spectrum (×100) of P3MT NW.

We investigated the nanoscale solid PL characteristics of the P3MT and the P3MT/Au NWs using color CCD and LCM system with an identical experimental condition. Figure 4a,b is PL color CCD images of a P3MT NW and a hybrid P3MT/Au NW, respectively. A weak emission from the isolated P3MT NW was observed, which could be explained by the doping effect (Figure 4a). It is well known that PL in highly doped conducting polymers is quenched due to the non-radiative recombination of exciton through polarons and bipolarons. After a while, after wrapping the P3MT NWs in Au, the emission of the P3MT/Au dramatically brightened, together with the red-shifted emission wavelength (Figure 4b). Figure 4c,d shows the images of three-dimensional (3-D) LCM PL intensity measured from an isolated P3MT NW and P3MT/Au NW, respectively. The Y-axis is the measured voltage of the sensor corresponding to the PL intensity. The measured voltage in the pristine P3MT NW (Figure 4c) was 7~9 mV, whereas it was 1.1~1.3 V in the P3MT/Au NW (Figure 4d). This is two orders higher than that of P3MT NW. The full spectrum of PL on P3MT/Au NW is shown Figure 4e, in which the black line is the spectrum of P3MT NW. The inset figure of Figure 4e is an enlarged PL spectrum of P3MT NW. Two emission peaks at around 640 nm and 685 nm in the P3MT/Au NW were clearly observed, which is in contrast with the single PL peak at around 544 nm in the P3MT NW. The two emission peaks of P3MT/Au NWs were at 96 nm and 141 nm—red-shifted compared to the maximum PL peak at 544 nm of P3MT NW, respectively—and the intensity showed a 170-fold enhancement compared with the maximum intensity of the P3MT. The cause of PL enhancement might be due to the Au wrapping the surface of the P3MT NWs with a thickness of 10 nm, which leads to strong enhancement of the radiative decay rate of the P3MT NWs through the resonant coupling of surface plasmons of Au with excitons in the P3MT NWs.

### 3.3. UV/Vis Absorption and Quantum Yield

**Figure 5:**
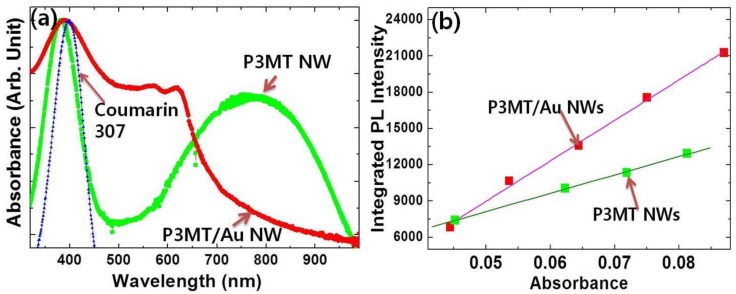
(**a**) UV-visible absorption spectra of P3MT/Au NWs and P3MT NWs. The dotted black line is the absorbance of Coumarin 307 shown as reference for the quantum efficiency; (**b**) The plot of integrated PL intensity vs. UV/Vis absorbance of P3MT/Au NWs and P3MT NWs for quantum yield.

We measured UV/Vis absorption spectra and PL quantum yield to prove the SPR coupling PL enhancement in the coaxial-like P3MT/Au NWs. Figure 5 shows the comparison between the normalized UV/Vis absorption spectrum of P3MT/Au NWs and that of P3MT NWs. A π-π* transition peak appeared at ~383 nm and a broad bipolaron peak was observed at ~800 nm in the P3MT NWs [36]. This indicates that the P3MT NWs are in highly doped states, which supports the conclusion that the PL of P3MT NWs were quenched due to the polarons and bipolarons. Meanwhile, after Au wrapping the doping-induced polaron and bipolaron peak was obviously suppressed and two new absorption peaks at ~567 nm (~2.19 eV) and ~616 nm (~2.01 eV) appeared. This means that the new energy bands appeared between HOMO and LUMO of P3MT, which should be at the interface between the Au metal and P3MT. This might be due to charge transfer through Fermi level alignment while making metal/polymer junction between Au and P3MT [37]. The Fermi level alignment induces band bending which causes the charge transfer from metal to the polaron/bipolaron states. This process leads to new visible sub-bandgap absorptions. It should be noted that the surface plasmon resonance of Au is in the visible range, which should be closely related to the two new absorption peaks of the P3MT/Au NWs (at around 567 nm and 616 nm) [38]. This strongly implies that the SPR of cladding-shaped Au could couple with the excitonic dipoles of P3MT, resulting in the energy transfer between the cladding-shaped Au and the core P3MT. Accordingly, we suggest that the SPR coupling in the hybrid P3MT/Au is the only cause of the extraordinarily enhanced PL. Additionally, it is known that core-shell nanostructures can have multiple plasmonic resonances [39], which correspond to the two new peaks of UV-vis absorption spectrum in Figure 5a. The resonance coupling modes provide the emissive decay of excitons, resulting in enhanced quantum efficiency.

To probe the PL efficiency of P3MT NWs with/without Au cladding, we estimated the quantum yield ($\phi_{QY}$) based on the comparison of the integrated PL intensity (Figure 5b). The experiment was undertaken using an excitation wavelength of 400 nm with P3MT/Au NWs dispersed in CHCl_3_. As a reference material for the measurement of $\phi_{QY}$, we used Coumarin 307 dissolved in methyl alcohol. Because the Coumarin 307 material has almost the same absorbance band as seen in Figure 5a and a high $\phi_{QY}\;$ (*φ_QY_* = 0.89). In Figure 5b, the increment of integrated PL intensity in both of P3MT/Au NWs and P3MT NWs show a linear correlation with the absorption intensity. From the equation $\phi_{QY}=\phi_{ST}\left(Grad_{x}/Grad_{ST}\right)\left(\eta_{x}^{2}/\eta_{ST}^{2}\right)$ [40], where the subscripts *ST* and *X* denote the standard sample (Coumarin 307) and the synthesized NWs, respectively. *Grad* refers to the ratio of the integrated fluorescence intensity vs. the absorbance at the excitation wavelength, and the *η* is the refractive index of the solvent. As expected, the estimated $\phi_{QY}$ of the P3MT/Au NWs was ~0.13, four times higher than ~0.032 of the P3MT NWs.

**Figure 6:**
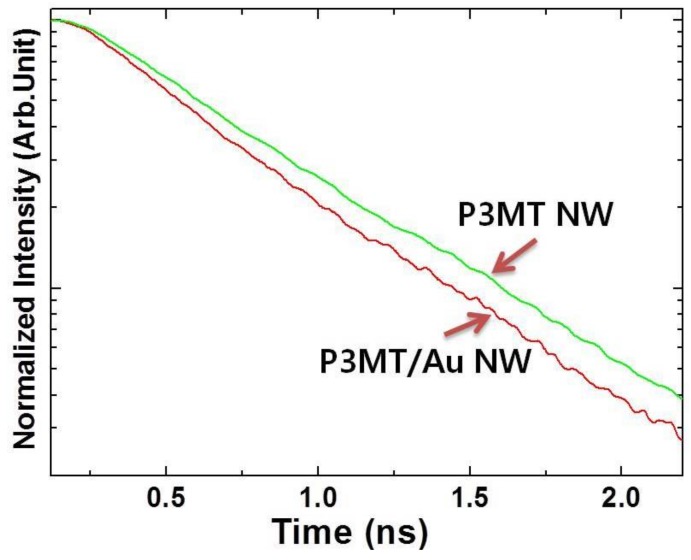
Time-resolved PL decay of P3MT/Au NWs and P3MT NWs.

To study the photoexcited exciton dynamics with surface plasmon effect, the PL decay lifetimes were measured by using the PCSTC system. The lifetimes were determined by nonlinear fitting curves based on the sum of two-exponential decay functions, *I_tot_ = A*_1_*exp*(*−t*/*τ*_1_) + *A*_2_*exp*(*−t/τ*_2_), where *I_tot_* is the integrated total intensity of PL. The average PL decay lifetime (*τ_ave_*) can be defined as *τ_ave_ = (A*_1_*τ*_1_ *+ A*_2_/*τ*_2_)/(*A*_1_ + *A*_2_) [41]. As shown in Figure 6, the average lifetime were about 410 ps for P3MT/Au NWs and about 480 ps for P3MT NWs measured at emission wavelength of 640 nm and 544 nm, respectively. The lifetime difference is, however, not enough to show the surface plasmon effect because of the fluorescence characteristics with a short lifetime of P3MT NWs, the shortening lifetime suggests that the PL enhancement of P3MT/Au NWs was induced by the enhanced radiative decay mechanism of excitons. This supports the conclusion that strong SPR coupling is deeply involved in this system.

### 3.4. Cross-Junction of P3MT/Au NWs

**Figure 7:**
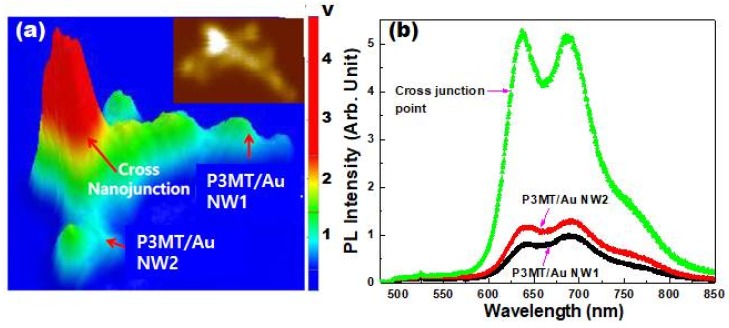
(**a**) 3-D LCM PL image of the overlapped P3MT/Au NWs. Inset: the mapping image of integrated PL intensity of the overlapped P3MT NWs; (**b**) Comparison of PL spectra measured between the cross-junction point and single NWs.

So far, we have probed the optical properties of the coaxial-like structured P3MT/Au NWs, in which the PL was observed from an individual NW. In general, the PL shows a linear increment as the number of emissive layer or amount of materials increases. However, we knew that there was a singular point in the PL intensity at which the heterostructured P3MT/Au NWs overlapped each other. Figure 7a is a representative PL mapping image observed from the overlapping structure of P3MT/Au NWs. The PL intensity is visualized as a 3-D LCM PL image with the unit of voltage corresponding to the PL intensity. It is interesting that the PL intensity of the cross-junction point was abnormally high even if considering the number of overlapped wires, which contrasted with that from the remaining area of NW1 and NW2. Moreover, this was ubiquitously observed throughout the whole sampling area. The inset of Figure 7a showing the integrated PL intensity also demonstrates that the cross-junction point is much brighter than other parts. The maximum value of measured voltage on the cross-junction point was 4.1–4.9 V, whereas that on the P3MT/Au NW1 and NW2 was 1.2–1.4 V. Figure 7b shows a comparison of the full PL spectra measured from the cross-junction point and the other NW area. The shapes of the PL spectra are similar. However, the PL intensity at the cross-junction point is more than three times higher compared with that measured from isolated P3MT/Au NWs (Figure 7b). The strongly enhanced field between the Au nanostructures fabricated like bowties induced molecular PL enhancement followed by the increment of quantum efficiency. The cross-junction point is analogous to the bowtie structure. Accordingly, the overlapping of P3MT NWs provides the synergy effect that the strongly enhanced field on the emissive decay of exciton. This might lead to the further enhancement of PL along with the resonance between the localized surface plasmons of the cladding Au and the absorption wavelength of P3MT [42,43].

## 4. Conclusions

We reported giant enhancement of the photoluminescence characteristics of coaxial-shaped heterostructures of P3MT/Au NWs. The cladding-shaped Au thin film wrapping the core-shaped P3MT NWs comprising the metal/polymer heterojunction induced the suppression of the bipolaron band. Additionally, the Au thin cladding extracted an extraordinarily strong PL, which might be due to the resonance of surface plasmonic modes of Au with the sub-band absorption of P3MT. Furthermore, the heterostructure of P3MT/Au NWs shows multiple plasmonic resonance modes. Another interesting point is that the cross-junction point of the P3MT/Au NWs shows an abnormal enhancement of PL intensity. This result shows one representative light-matter interaction in nanoscale at the interface of metal and polymer, where the surface plasmon resonance occurs and induces the non-linear optical properties. Consequently, we demonstrate a way of studying peculiar phenomena only observed in nano-optics that can be applied to improving otpoelectronic devices.
